# Cobalt–Silicon Material Libraries Derived from High Throughput Experimentation and Their Application in Lithium Batteries

**DOI:** 10.1021/acsomega.5c02694

**Published:** 2025-10-01

**Authors:** Sven Uhlenbruck, Sandra Lobe, Yoo Jung Sohn, Christian Dellen, Walter Sebastian Scheld, Dina Fattakhova-Rohlfing, Olivier Guillon

**Affiliations:** 1 Institute of Energy Materials and Devices, Materials Synthesis and Processing (IMD-2), 28334 Forschungszentrum Jülich GmbH, Jülich 52425, Germany; 2 Helmholtz Institute Münster: Ionics in Energy Storage (IMD-4), Forschungszentrum Jülich GmbH, Corrensstr. 46, Münster 48149, Germany; 3 Faculty of Engineering and Center for Nanointegration Duisburg-Essen (CENIDE), Universität Duisburg-Essen, Lotharstraße 1, Duisburg 47057, Germany; 4 Jülich Aachen Research Alliance: JARA-Energy, Jülich 52425, Germany

## Abstract

Silicon is one of the most promising anode materials in lithium batteries due to its high storage capacity; however, it suffers from extensive volume changes during charge and discharge in a battery cell. Mixtures of silicon with transition metals potentially forming transition metal silicides are under intense investigation as mechanically stable and electronically conductive frameworks with additional silicon in between. The overall capacity for lithium storage in such mixtures in general decreases with increasing transition metal content, and the interpretation of the data in the literature is controversial. In this work, it is shown how approaches toward high-throughput experimentation allow a deeper insight into the details of phase formation in transition metal silicides and thus their impact on the electrochemical performance of silicon-silicide frameworks as electrodes for advanced lithium batteries. Silicides appear to evolve solely in narrow compositional ranges around commensurate ratios of cobalt and silicon, while the observed capacity reduction is attributed to an impeded and finally blocked motion of lithium ions.

## Graphical abstract

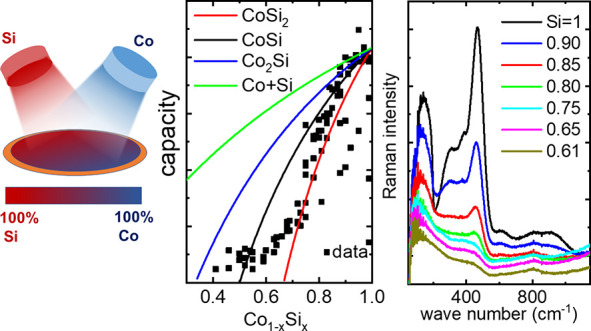

## Introduction

Silicon features one of the highest capacities for lithium ions, ∼3.500 mAh g^–1^, but it suffers from tremendous volume changes during charge and discharge. Moreover, the low lithium ion conductivity in silicon and the formation of solid-electrolyte interphases (SEIs) are significant barriers to the application of silicon as an electrode material in lithium batteries.^1–3^

Transition metal silicides (in particular with cobalt (Co), nickel (Ni), titanium (Ti), and copper (Cu)) have been thoroughly investigated as potential alternatives or at least as scaffold structures, mixed with silicon, for example, made by a series of sputter-deposited metal–silicon mixtures with no additional external heating (see, for example,^4–6^ and references therein). Another advantage of silicides is their comparatively high electronic conductivity and thermal stability, which paved their way to applications in microelectronic components and devices.^7–9^

The experimental findings^4–6^ showed in general a continuous decrease of the measured capacity of transition metal–silicon mixtures with increasing transition metal content. It was discussed that the metals and silicon may have reacted to metal silicides with commensurate ratios: MSi_2_, MSi, and M_2_Si (with M as transition metal and Si as silicon) once both constituents are present. Moreover, the different publications came to different conclusions about their contribution to the electrochemical behavior: either electrochemically active or inactive.

In this work, a series of cobalt–silicon mixtures prepared by magnetron sputtering were composed as a so-called “compositional gradient”, which means that a higher amount of material from one sputter target is deposited on one side (which is closest to the target) of a comparatively big substrate and less material at the opposite side, and vice versa for a second target. X-ray diffraction and Raman spectroscopy showed that cobalt addition significantly affects the short-range order in silicon and that the CoSi_2_ and CoSi commensurate phases only occur in very narrow ranges of composition. Similar to previous scientific work on this topic, a general trend to lower overall capacity for lithium with increasing cobalt content was observed here; however, the data suggest that the capacity reduction with increasing cobalt content is independent of the formation of cobalt silicides.

## Experimental Section

Thin film depositions were carried out in a physical vapor deposition (PVD) cluster system CS400S manufactured by Von Ardenne, with an argon gas filled glovebox attached to the load lock chamber. Co–Si thin films were codeposited via radio frequency (rf) magnetron sputtering in an Ar plasma at 5 × 10^–3^ mbar pressure during deposition along with a gas flow rate of 30 standard cubic centimeters per minute in a confocal arrangement of two targets, a silicon target (99.999% purity; FHR, Ottendorf-Okrilla, Germany) and a cobalt target (99.95% purity; FHR, Ottendorf-Okrilla, Germany), opposite to each other. The sputter power was varied between 0.5 and 8 W cm^–2^ for Si and 0.5 and 5 W cm^–2^ for Co, no external bias power was applied, and sputter times were varied from 1200 to 10,800 s. The base pressure prior to deposition was below 3 × 10^–7^ mbar, and the argon gas for sputtering had a purity of nominally 99.999% and a typical oxygen content of about 1 ppm. A PVD coating time of 2 h with sputter powers of 6.4 W cm^–2^ for Si and 0.9 W cm^–2^ for Co led to a coating thickness of about 1 μm, with an increase close to the part with almost pure Si. Commercial battery grade copper foil with a thickness of nominally 0.009 mm, supplied by MTI, and (001) silicon wafers with a PVD copper coating (mono pulse sputtering from a metallic copper target (99.99% purity; FHR, Ottendorf-Okrilla, Germany) with 200 W power for 1 h, leading to a thickness of 800 to 900 nm) were used as substrates for Co–Si deposition. A thermo couple close to the substrate was used to continuously monitor the temperature of the substrate during Co–Si sputtering, since an unintentional heating could in principle occur due to the heat impact of the sputter plasma to the substrate and so-called condensation heat of the condensing particles from the gas phase. The measured temperatures were logged by the PVD system over the entire deposition time. They never exceeded 40 °C. Copper foil punched into discs of ∼12 mm diameter was used for electrochemical cell tests in 2032 coin cells, also to avoid any contribution of silicon of the wafer to the electrochemical measurement. Copper is known to ingest small amounts of lithium; however, Rupp et al. showed that this effect can be neglected for typical time scales of battery cell operation.^10^ The coated Si wafers were used for some analytical measurements where planar surfaces were required, for example, quantitative energy-dispersive X-ray spectroscopy (EDS) [the copper foil had corrugations from manufacture (rolling), and some coated foils bent due to typical compressive stresses in the PVD layers]. The PVD copper layer between the Si wafer and cobalt silicides was introduced to mimic best the deposition conditions on copper foil and to avoid a contribution of the silicon of the wafer to the electrochemical reaction with lithium. All substrates were Ar sputter etched prior to PVD deposition to remove any residual contaminations.

Scanning electron microscopy (SEM) along with energy-dispersive X-ray spectroscopy (EDS) was performed on samples that were sputter-coated with a thin platinum layer (several nm) by employing a Zeiss GeminiSEM 450 (Carl Zeiss Microscopy Deutschland GmbH) device equipped with an Ultim Max 170 EDS detector (Oxford Instruments) and a Zeiss Evo 15 SEM (Carl Zeiss Microscopy Deutschland GmbH) device equipped with an Ultim Max 100 EDS detector (Oxford Instruments). A beam accelerating voltage between 15 and 20 kV, the latter one for the proper detection of copper, was used. The samples were measured versus internal standards. X-ray diffraction data were obtained with a Bruker D4 Endeavor device using Cu–K_α_ radiation (Bruker AXS GmbH) and an Empyrean instrument (Malvern Panalytical GmbH) in a grazing incidence geometry, employing Cu–Kα radiation. The elemental composition of each electrochemically tested sample was assessed by optical emission spectroscopy with an inductively coupled plasma (ICP-OES), employing an iCAP7600 spectrometer (Thermo Scientific). Prior to the spectroscopy measurements, the samples were ground in a boron carbide mortar, and 50 mg of each sample was subsequently melted with sodium borate at 1050 °C for 30 min. The melt was dissolved in 30 mL of hydrochloric acid (5% concentration). The elemental ratios of cobalt and silicon derived from ICP-OES were rated to show the actual ratios of cobalt and silicon within an accuracy of 4%. The SEM/EDS analysis results corroborated this assessment. Confocal white light topography (CT 350 T, cyberTECHNOLOGIES GmbH, Eching, Dietersheim, Germany), glow-discharge optical emission spectroscopy (Profiler 2, HORIBA Jobin Yvon GmbH, Oberursel, Germany), and electron microscopy were employed to assess the uniformity and comparability of the coated samples. Raman spectra were obtained on both types of samples (copper foil and copper-coated silicon wafer) by employing a Renishaw InVia Raman microscope equipped with solid-state lasers (532 and 785 nm), an objective lens with 50× magnification with a large working distance of ∼7.5 mm and an aperture of 0.55, and a grating of 2400 lines per mm and 1800 lines per mm, respectively. The measured area was 75 μm by 40 μm with a step size of 1 μm. The laser power was set to 5 mW to avoid the danger of laser-induced sample damage. A total of 3.000 spectra were obtained per sample with an acquisition time of 1 s per spectrum. The spectra were truncated to the range from 50 cm^–1^ to 1250 cm^–1^. The cosmic rays of the raw data were removed with a built-in function of Wire 5.2 software (Renishaw). Afterward, the data sets were averaged by using the averaging procedure within the Wire 5.2 software (Renishaw). Unless otherwise stated, the X-ray and the Raman data, respectively, were collected from a Co–Si PVD layer on a Si wafer with a PVD copper layer, deposited with the following deposition parameters: 2 h deposition time with sputter powers of 6.4 W cm^–2^ for Si and 0.9 W cm^–2^ for Co.

All electrochemical tests were performed with round-shaped cells in commercial 2032 coin cell housings. PVD Co–Si electrodes on copper foil were measured versus lithium metal. The lithium foil, delivered by Alfa Aesar/Thermo Fisher, had a thickness of 0.75 mm, with a purity of 99.9% (metals basis). The electrolyte was 15 wt % fluoroethylene carbonate (FEC) in 1 M lithium fluorophosphate (LiPF_6_) in ethylene carbonate (EC)/ diethyl carbonate (DEC) (v:v = 1:1). FEC (99% purity) and 1 M LiPF_6_ in EC/DEC (battery grade) were purchased from Sigma-Aldrich. These two compounds were mixed to obtain 15 wt % FEC in 1 M LiPF_6_ in EC/DEC. Amounts of 200 to 260 μL of liquid electrolyte were used in each cell. A sandwich of a Celgard Li-ion battery separator film with 25 μm thickness and 20 mm diameter and a Whatman GF/D separator with 15 mm diameter was used. The coin cell setup was as follows: “anode side” coin cell housing, a spacer of 0.5 mm, lithium foil, Whatman separator, Celgard separator, sample, a spacer of 1.0 mm, spring, “cathode side” coin cell housing.

All cells had three formation cycles with a current density of 20 μA cm^–2^ between 1.2 and 0.05 V. Supplemental impedance spectra of the cells were recorded prior to and after the formation process by a VMP 300 potentiostat (Biologic). Charge–discharge cycling was performed with a MACCOR Series 4000 (Maccor Inc.) along with a range of charge/discharge rates (0.1C, 0.5C, 1C, and 5C, where C denotes a rate in which the nominal capacity of the electrode is completely charge/discharged in 1 h).

It is important to accurately measure the PVD film thickness for calculation of the theoretical specific capacity. Therefore, the PVD layer thickness was also assessed by a combination of SEM and glow discharge optical emission spectrometry (GD-OES).

## Results and Discussion

It can be estimated from the system parameters during PVD like base pressure, deposition rate, deposition time, and flow rate as well as the purity of the argon gas that there is virtually no oxygen present compared to the amount of cobalt and silicon atoms deposited; thus, no noteworthy oxidation of the material could occur during sputtering. This is important for the later discussion of the electrochemical data since cobalt oxides and silicon oxides would significantly impact the theoretical capacity of the electrode.^11–14^ In addition, samples were exposed to ambient air for ∼100 h and subsequently checked by scanning electron microscopy (SEM) along with energy-dispersive X-ray spectroscopy (EDS) and Raman spectroscopy: no changes with regard to microstructure, composition, or phase could be resolved; i.e., the samples would not change their key properties even in the event of a small exposure to air.

Figure 1 summarizes the resulting capacities of cobalt–silicon mixtures extracted from the electrochemical measurements of 152 samples (coin cells). Though the C rates/the current densities were varied by a factor of 50, the majority of the data points fit to the general trend shown in Figure 1; thus, the actual current densities during these experiments are apparently not a crucial factor.

**Figure 1:**
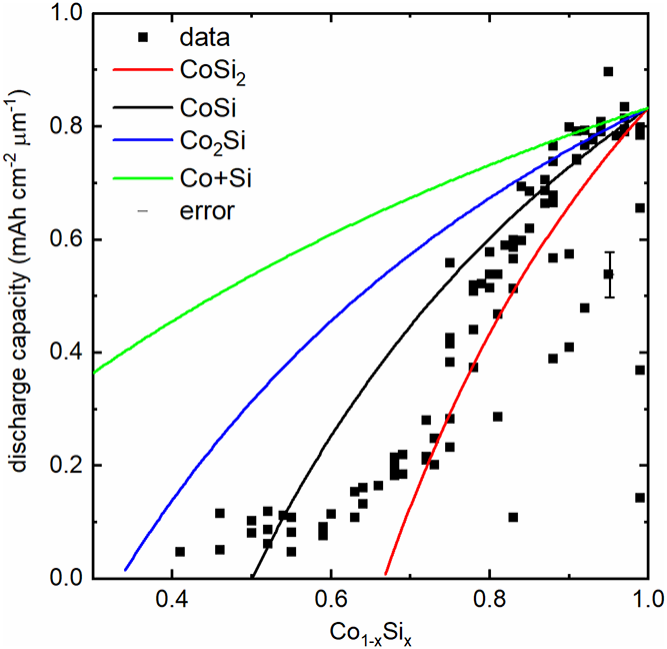
Initial discharge capacity of cells comprising cobalt–silicon mixtures and lithium metal, normalized to a 1 cm^2^ area and 1 μm thickness (lithiation of the cobalt–silicon mixtures). For Si, 1 mAh cm^–2^ μm^–1^ would be mathematically equivalent to 4280 mAhg^–1^. The maximum value of about 0.82 mAh cm^–2^ μm^–1^ at *x* = 1 corresponds to the specific capacity of ca. 3500 mAhg^–1^ of Si. The measured capacity values were rescaled (multiplicated) by a factor 1.12 to match the theoretical capacity of pure silicon (*x* = 1). The lines denote theoretical capacities assuming that cobalt/cobalt silicides were electrochemically inactive, and only the remaining silicon was electrochemically active. The largest errors in the capacity data appear to mainly stem from measurement uncertainties of the geometry of the electrodes, which is shown as an example with the error bar at one data point.

The results match well to the findings in previous publications, in which the reduction of the capacity values were associated with the formation of cobalt silicides like CoSi or CoSi_2_.^5^ The initial capacity of all measured cells close to pure silicon electrode material appears to be around 12% lower than the theoretically possible capacity of silicon. One explanation could be some initial irreversible reaction, which has been described in the literature for silicon.^2^ Another explanation could be that PVD layers in general always exhibit some microporosity (see also Figure 7) and – depending on substrate defects – may have a few voids so that the assumption of a 100% dense anode layer overestimates the actual amount of silicon in the PVD layer. This is discussed in more detail later in the description and interpretation of the data within the framework of percolation theory.

Intentionally, *all* data points of the measurement matrix were plotted, though for a few cells, there apparently happened an error in the cell buildup, thus resulting in a small number of data points with larger deviations from the general trend.

The figure also contains lines that represent the theoretical maximal capacity assuming that either just mixtures of silicon and cobalt, CoSi_2_, CoSi, or Co_2_Si was formed in the PVD-deposited layers, and pure cobalt metal^15^ and the formed silicides were electrochemically inactive. For CoSi, for example, it is clear that – from theory – the capacity would be zero for *x* = 0.5, since all silicon would have been consumed for CoSi. (The slight curvatures are due to the fact that the electrode volume in the graph is fixed, while cobalt, silicon, and the silicides differ in their molar volumes.)

At first glance, it suggests itself to associate the decrease in capacity with the increase of cobalt silicide amounts, as discussed in previous publications. However, there are three distinct features that do not fit this hypothesis. First, the slope of all the theoretical curves for silicides starts at Si = 100% with a rather large slope, while the experimental data show a low, almost no slope for the range of Si close to 100%, followed subsequently by a rapid change in slope. Second, the theoretical curves exhibit no changes in the curvature, while the experimental data demonstrate a clear change in the curvature (similar to a stretched character “S”, therefore in the following called “S shape type”), in agreement with the data of previous publications.^4–6^ Third, if Co and Si would completely react to Co_2_Si, CoSi, and CoSi_2_, and if these reaction products are not electrochemically active, there should be zero capacity at *x* = 1/3, 1/2, and 2/3, respectively; however, there is a clear residual capacity measured for all samples. An artifact due to measurement errors can be excluded, according to the accuracy specifications of the electrochemical test system – the equipment does indeed measure finite capacities. The following sections will show that only small portions of Co and Si react to cobalt silicides and do so only in a narrow compositional range close to *x* = 1/3, 1/2, and 2/3 for Co_1–*x*_Si_*x*_.

Raman analysis was performed to gain more insight into the details of the lattice bonds in the cobalt–silicon mixtures. In a stricter sense, such Raman spectroscopy observes an inelastic scattering of light at lattice vibrations of crystal phases/structures. In the following, signals of inelastic scattering in amorphous crystal structures, especially amorphous silicon, are also discussed. For the sake of compatibility to the existing scientific literature of cobalt silicides, and better readability of this work, such signals will be also called “Raman” signal, though it does not correspond to solid-state Raman spectroscopy in the stricter sense.

Figure 2 shows the Raman spectra of the cobalt–silicon mixtures on copper-coated Si wafers in the compositional range described in Figure 1. Pure silicon exhibited the strongest signal. The shape is in agreement to previous publications of experimental and theoretical Raman signals in amorphous silicon.^16–18^ The signals of all four Raman “modes” of pure amorphous silicon, indicated by arrows in Figure 2a, were immediately and significantly reduced upon addition of cobalt, most pronounced for the signal between 450 and 500 cm^–1^, which has previously been described as a “transversal optical mode”.^18^ (Note that the Co–Si Raman curves were neither shifted nor background subtracted.) Raman scattering is a probe for lattice vibrations and, thus, for lattice bonds. Therefore, this damping of Raman signals in amorphous silicon is already an indication that the lattice bonds in silicon are already affected by low cobalt addition. It is noteworthy that there is no indication for a crystallized cobalt silicide, CoSi, in the compositional range of 100 at % Si to 61 at % Si, while the main Raman peak of CoSi at 204 cm^–1^ ^19^ can be identified in a very confined region around *x* ≈ 0.5. (Figure 2b, blue rectangle, and Figure 3). This means that a kind of long-range order crystallization of CoSi occurred in this region, but not for other compositions, especially not in the area of high silicon and low cobalt content. Moreover, three novel peaks appear in the compositional range between 61 at % and 37 at % (marked with “v” in Figure 2b). According to Longhin et al.,^20^ there are no Raman peaks of CoSi for wavenumbers higher than 430 cm^–1^, and CoSi_2_ is not Raman active.^21^ Therefore, the origin of these peaks is not yet known.

**Figure 2:**
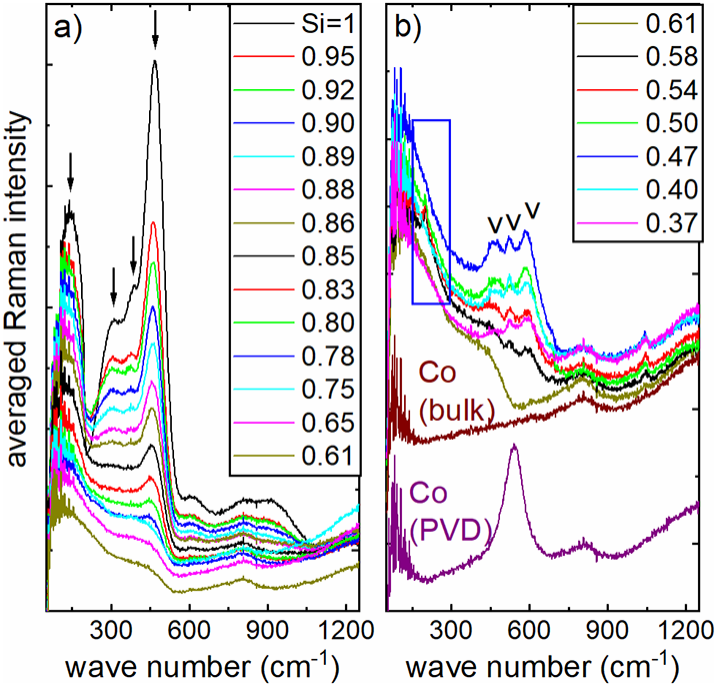
Averaged Raman signal intensity for cobalt–silicon mixtures on a copper-coated silicon wafer. The numbers in the graphs specify the silicon content in fractions of 100 at %. Graph (a): 100 at % Si to 61 at % Si; graph (b): 61 at % Si to 37 at % Si. The curves of Co (bulk) and Co (PVD) were shifted downward for clarity. The arrows in (a) mark positions of optical modes in amorphous silicon as published in the literature (see text). The blue rectangle shows the region where the main peak of CoSi is located. A magnification of this region is shown in Figure 3. Unknown peaks in (b) are tagged with “v”. Si fractions were derived from EDS measurements.

**Figure 3:**
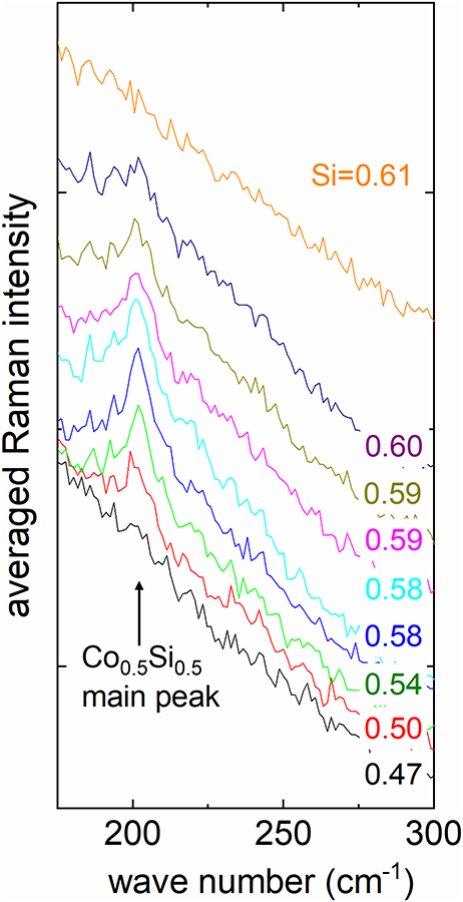
Magnification of Figure 2, adapted, extended with more original Raman data, in the region around the main Raman peak of cobalt silicide, CoSi, at 204 cm^–1^. Si content percentages were rounded to two significant digits; therefore, there are double occurrences of the same numbers though the analyzed material may in fact have slightly different composition. The curves were slightly shifted for the sake of clarity. Si fractions derived from EDS measurements.

The Raman spectra of a massive piece of metallic cobalt (“Co (bulk)”) and of a PVD layer of pure cobalt (“Co (PVD)”) are also included in Figure 2b. Obviously, the Raman signals at higher wavenumbers are by the majority influenced by inelastically scattered light of cobalt. On the one hand, cobalt has a hexagonal closed package crystal structure, not a monatomic Bravais lattice; therefore, optical vibrations are in principle possible. On the other hand, it may be difficult to excite such Raman-active vibrations since metals in general do not allow visible light to go a few hundreds of nanometers into the structure but reflect the light mainly elastically like a mirror, especially in close to a so-called “180° geometry”, where the incoming light beam, mainly perpendicular to the sample surface, and the reflected/scattered light have an angle near 180°. Of course, there are some deviations due to the confocal arrangement; an angle of 180° is a fair approximation with a long focus/working distance of 7.5 mm and an aperture of 0.55 between objective lens and the samples. The measurement was performed with two different laser excitation wavelengths, and the result could be reproduced. This gives rise to the assumption that the signal is not due to the fluorescence of the sample. According to the scientific literature, cobalt allows electronic excitations from the valence band,^22^ and Raman-like signals were obtained for cobalt nanoparticles^23^ and cobalt metal dimers,^24^ so that in general inelastic light scattering may be possible. A deeper analysis of this is, however, beyond the scope of this work.

With regard to the intensities of the Raman, it can be assumed that only a small portion of the cobalt–silicon mixture crystallizes to silicides.

In addition to Raman analysis, X-ray diffraction measurements of the thin films were performed also to look for potential CoSi_2_ phases, which were not visible in Raman spectroscopy.

In Figure 4a, there are five clear reflections in the diffraction pattern that match well with copper, presumably from the underlying copper layer. Moreover, a broader feature around ∼30° 2 theta (marked with a black arrow) and, similarly, around 52° and 85° 2 theta is found. These are roughly the positions where a signal of crystalline silicon (ICDD PDF no. 01–070–5680) would be expected. With decreasing silicon content, these features gradually decrease, too. It is therefore concluded that these marks are associated with a slight indication of long-range order in the crystal structure of the silicon in the thin film. Due to the grazing incidence diffraction geometry and the crystal orientation of the wafer, it is assumed that this silicon signal does not stem from the silicon substrate beneath the copper layer.

**Figure 4:**
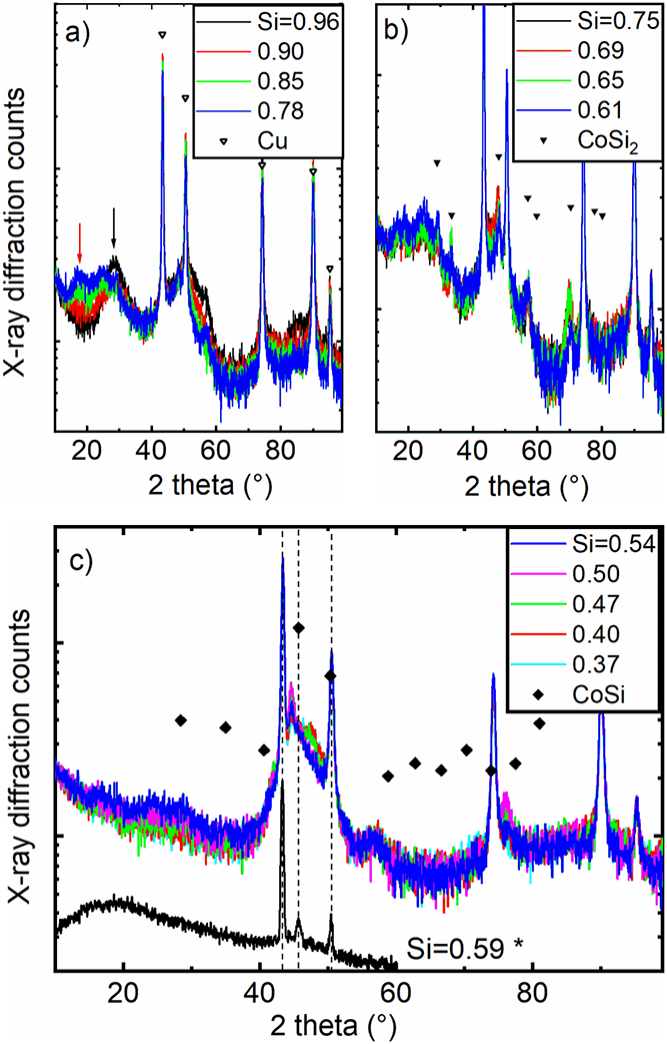
Overview of the X-ray diffraction results of Co–Si mixtures on a copper-coated Si wafer (exception: the curve with an asterisk * (Si = 0.59) was Co–Si on copper foil). The numbers in the graphs specify the silicon content in fractions of 100 at %.The black arrow in (a) denotes the position of silicon, the red arrow denotes an unidentified reflex position (see text). The open triangles show the reflection positions of copper (International Centre for Diffraction Data (ICDD), Powder Diffraction File (PDF) no. 01–070–3039). The triangles in (b) denote the position of CoSi_2_ (ICDD PDF no. 03–065–8980), and the diamonds in (c) mark the Bragg reflections of CoSi (ICDD PDF no. 01–072–1328). The dotted lines in (c) are a guide to the eye for the positions of the three copper reflexes. The *y* axes are in a logarithmic scale. Si fractions were derived from EDS measurements.

It cannot be answered if also first indications of a long-range order of cobalt are present since the reflection positions of cobalt (ICDD PDF no. 00–005–0727) are very close to those of copper and are likely superimposed/hidden.

In addition, a reflection arises at low angles (marked with a red arrow); the origin, however, has remained unclear at this stage.

A set of novel reflections appear once the composition approaches *x* = 2/3: Figure 5 shows how the reflections evolve and again vanish with composition in the small compositional range of *x* = 2/3 (which corresponds well to the main reflection of CoSi_2_). A similar behavior can be found for the other reflections of CoSi_2_ around 28 and 35° 2 theta.

**Figure 5:**
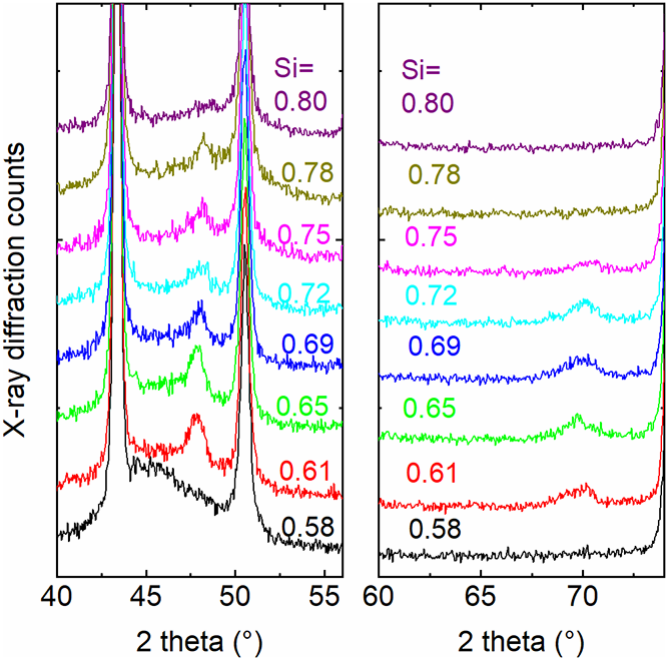
Magnification of Figure 4b, and more diffraction patterns added, around 48° 2 theta and 70° 2 theta. The *y* axes have a linear scale, and curves are slightly shifted to each other for clarity. Si fractions derived from EDS measurements.

Figure 6 shows an analogous analysis for *x* values around 0.5 close to 45 and 76° 2 theta, where another set of reflections appear that match well to the pattern of CoSi. The fact that not all reflex positions of the powder diffraction data of CoSi were found in the experimental data here is attributed to a preferred orientation of CoSi.^25^ With regard to the intensities of the reflections, it is reasonable to assume that only a small portion of the mixtures crystallize to silicides in the sense that it can be detected by X-ray diffraction.

**Figure 6:**
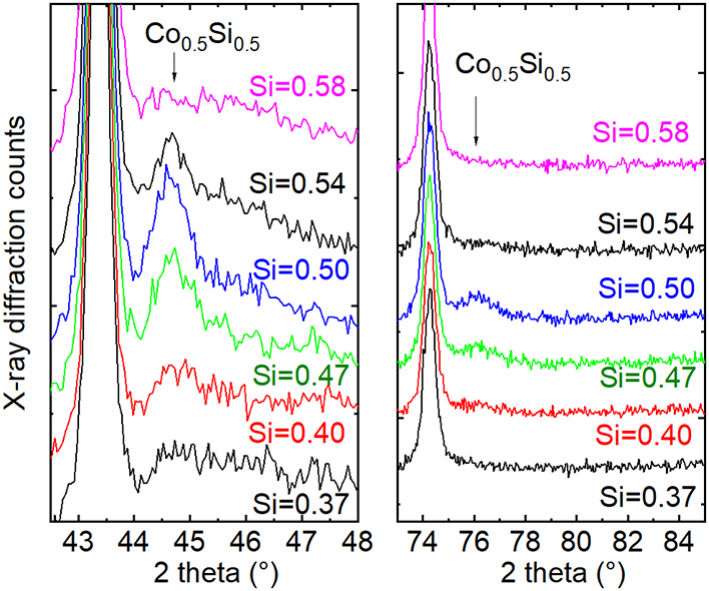
Magnification of Figure 4c, and more diffraction patterns added, around 45° 2 theta and 76° 2 theta. The *y* axes have a linear scale, and curves are slightly shifted to each other for clarity. Si fractions derived from EDS measurements.

In addition to the X-ray diffraction pattern on a copper-coated Si wafer, a pattern of cobalt–silicon mixtures on a copper foil is shown in Figure 4c (black curve). The X-ray diffraction measurement on copper foil was intentionally made on the diffractometer of Bruker, while the other measurements were performed with the device of Panalytical: since the reflection positions of copper fit to each other, it can be excluded that there has been an artifact by the equipment. While the reflection position of copper matches mainly exactly for both cases, silicon and copper substrates, there is a slight shift for the position of the CoSi feature in the case of cobalt–silicon mixtures on a copper-coated wafer, while it matches well for the coating on a copper foil as the substrate. Such deviations in the reflection position of CoSi are also published in the literature.^25–27^

For both cases, CoSi_2_ and CoSi, it is obvious from these experiments that CoSi_2_ and CoSi crystal phases exist only in a small compositional range (1:1 and 1:2, respectively) of cobalt–silicon mixtures applied under the chosen PVD conditions. This phase development is in agreement to the published general phase diagrams,^28^ where thermodynamic analyses show that the silicides CoSi_2_ and CoSi are not formed at relative Co contents between 0 and 0.7. Thus, these silicides cannot be taken into consideration for an explanation of the capacity reduction with increasing Co content in Co–Si mixtures. Different preparation conditions in general may have an impact on the phase development; for example, a higher substrate temperature is supposed to lead to a higher degree of crystallization of both the silicide phases and the (in this case amorphous) silicon layer. Still, CoSi_2_ and CoSi crystal phases solely exist in a small compositional range and cannot explain the capacity reduction shown in Figure 1.

The effect that phases with a couple of elements may only exist in a small compositional range with certain simple ratios of integer numbers is known as an *interlocking* into commensurate phases, which is associated with gain of overall lattice energy due to fewer stress/strain.^29–36^ In this context, it needs to be mentioned that other sources of mechanical stresses within the PVD layers may impact the onset or details of the phase developments. Such sources of stresses can be the deposition parameters during PVD, individual material properties like melting points or molar volumes, or the rigidity of the substrates (for example, flexible thin copper foil vs stiffer silicon wafer). This might be the reason for the slight deviations of the X-ray diffraction results within the presented experiments on different substrates and the different results in the literature that were discussed in the previous sections.

The X-ray diffraction and Raman data suggest that there are clear changes in the crystal lattice bond character within the cobalt–silicon mixtures with increasing cobalt content, but a crystallization of CoSi_2_ and CoSi does not occur at low cobalt concentrations (in the sense that it can be detected by methods sensitive to crystal lattices such as X-ray diffraction or Raman spectroscopy). Moreover, no indication was found about the formation of any other crystal phase that could account for the major suppression of the electrode capacity in the compositional region between 95 at % silicon and 70 at % silicon (Figure 1).

Therefore, a different description of the capacity decrease is now proposed from the experimental capacity characteristics shown in Figure 1, based on a transport description of heterogeneous media.

Figure 7 shows the microstructure of the deposited Co–Si layers as a function of the Si content. The microstructure differs significantly for different Co/Si ratios. For Si = 0.36, the morphology consists of densely packed columns with lateral dimensions in the order of magnitude of 10 nm, which is the typical morphology of metals deposited by PVD at low temperature according to the Thornton diagram. The microstructure changes to a more feather-like shape and then to a compact form with tilted columns. For the highest Si content, no particular textures can be identified, which might be an indication of an amorphous layer part.

**Figure 7:**
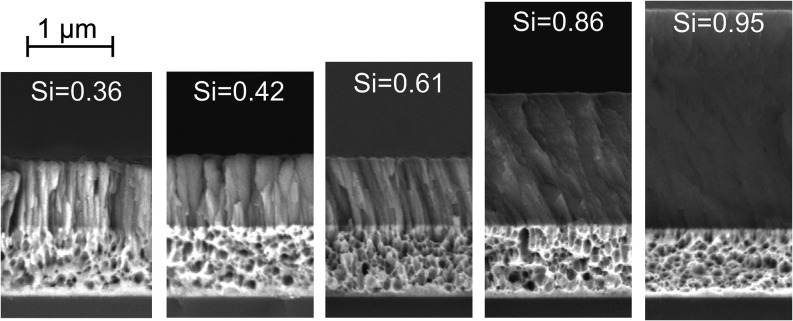
SEM images of fracture surfaces of a Si wafer coated with a 800–900 nm Cu layer and subsequently coated with a Co–Si gradient layer (2 h PVD coating time with sputter powers of 6.4 W cm^–2^ for Si and 0.9 W cm^–2^ for Co). Images were taken with backscattered electrons at 15 kV acceleration voltage.

It can be derived from the electrochemical tests that the electrolyte with the lithium ions is in close contact to the PVD layer since the cell measurements could be performed until rather high currents. The dimensions of the columns are orders of magnitude larger than the ionic radii of lithium ions; therefore, it is supposed that structural details of the columnar microstructure do not impact the general electrochemical behavior.

Lithium ions require a continuous network of conducting paths to reach all of the available lithium acceptance sites in the electrode. This is, of course, the case for nominally 100% silicon, even if a few voids or columnar structures are present. As long as the concentration of cobalt is low (also taking the different molar volumes of silicon, 0.08 mol cm^–3^, and cobalt, 0.15 mol cm^–3^, into consideration), the continuous network for lithium ion transport throughout the entire electrode layer is maintained, and the ions can be stored all over the Co–Si layer, as in pure silicon.

It is clear that this continuous network of silicon sites will break at a certain high amount of cobalt in the layer, and a continuous transport of lithium along the silicon network is impeded. In this compositional region, a more pronounced, maybe even sudden, drop in the capacity will occur due to the breaking of the network. With a greater increase of cobalt, the capacity is then mainly dominated by the capability of regions with high cobalt and small silicon content to store lithium ions – the curve may flatten again.

Such a behavior is captured by the so-called percolation model, which is a universal model for the description of transport phenomena in heterogeneous media. This behavior can be roughly compared with the conductance of a (insulating) beaker filled with small metal and glass spheres: while still mainly metal spheres are present, the conductance between the top and the bottom of the beaker is dominated by the metal spheres and only slightly reduced by addition of glass spheres (“range 1”). At a certain concentration of glass spheres, the so-called percolation threshold, however, the continuous conductive paths of the metal spheres are drastically reduced and finally eliminated, and the conductance between top and bottom of the beaker decreases rapidly (“range 2”) and then is mainly dominated by the conductivity of the glass spheres. With a further increase of the fraction of glass spheres, the overall conductance does not change notably any more (“range 3”). These three distinct ranges 1 to 3 in such a percolation model – a rather constant range, followed by a range with sudden steep change, followed again by a rather constant range – is regarded as the origin for the shape of the data curve shown in Figure 1.

The percolation thresholds for perfect three-dimensional lattices are in the range of 10 to 30% of conductive sites. However, it needs to be taken into account, that the micro- and nanostructures of the PVD layers are far away from a perfect crystal, which may impede the ion transport, for example, by micropores, and may effectively change the particular values for the percolation threshold, and the percolation threshold in general shifts to even higher values in the case of a directed percolation (in this case, as a consequence of an applied electrochemical potential).^37,38^ Though such structural details may lead to moderate changes in the actual percentage value for a particular percolation threshold, the general percolation model itself and its derivatives remain valid.

Electrochemical impedance spectroscopy (EIS) data taken after the formation procedure were correlated with the Si content (Figure 8). Figure 8a exemplary shows the EIS data of three parts of one PVD-coated Si wafer in a Nyquist plot. The shape of the curves is satisfactorily in accordance with the data in the literature.^39,40^ The upper end of a semicircle-like part of the collected data can be interpreted as a measure for the resistance of the setup.^41^ These values, denoted as Re­(Z)_i_, were extracted from sixty different PVD deposition runs, each with Co–Si compositional gradients (Figure 8b).

**Figure 8:**
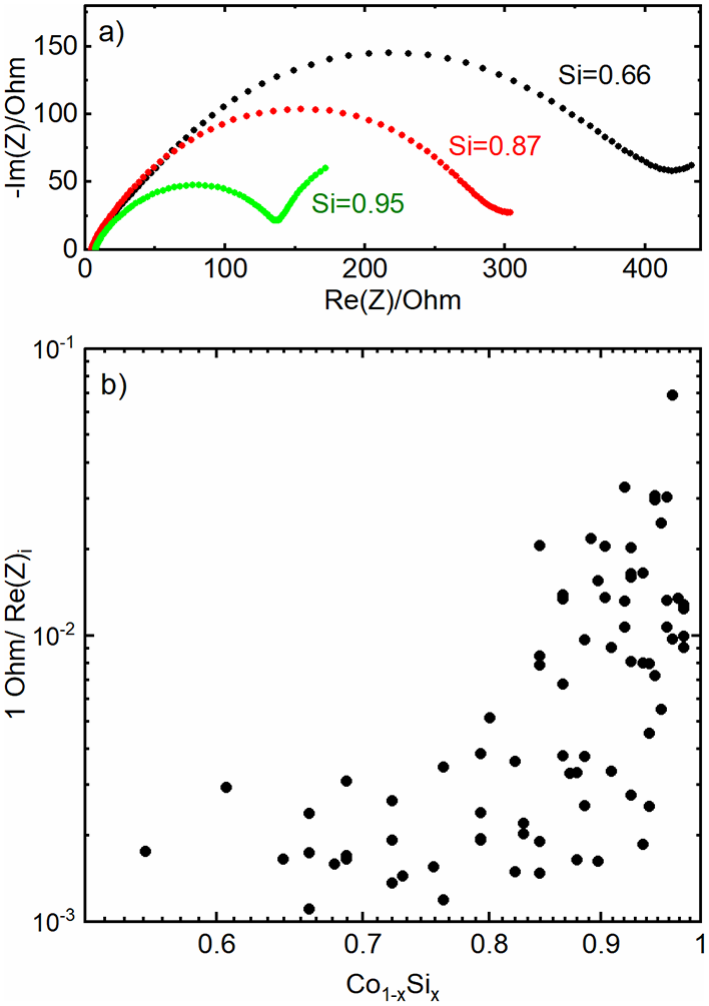
(a) EIS data of three selected samples with different relative Si volume contents in a Nyquist plot; (b) derived conductivity values 1/Re­(Z)_i_ from electrochemically analyzed samples.

An approximate estimation of a possible contribution of the silicon to the resistance was performed by utilizing the Nernst–Einstein equation *D*= μ*k*_B_ *T*/*q* and σ = *qn*μ, where *D* is the diffusion coefficient of Li in Si, μ is the mobility, *k*_B_ is the Boltzmann constant, *T* is the absolute temperature in K, *q* is the charge (1.6 × 10^–19^ C), σ is the conductivity, and *n* is the charge concentration. Assuming the experimental data *D* = 10^–11^ cm^2^/s (taken from Li et al.^42^), *T* = 300 K, *n* ≈ 4.8 × 10^22^ cm^–3^ (equivalent to one Li atom per Si atom (significantly less than maximum uptake, so that blocking effects due to a too high Li site occupancy do not need to be taken into account)), 1 cm^2^ electrode area, and 1 μm electrode thickness, a conductance in the range of 10^–1^ to 10^–2^ 1/Ohm can be evaluated. Therefore, it can be assumed that the silicon-based electrode has a significant impact on the conductance data in Figure 8 due to the low diffusion coefficient *D* of lithium in silicon.

The conductance of Co–Si mixtures is extremely reduced with increasing Co content, approximately with a power law of *x* ^8^, derived from the linear least-squares fit of log_10_(1 Ohm/Re­(Z)_i_) versus log_10_(*x*) in the range of *x* = 0.7 to *x* ≈ 1 in Figure 8, with *x* as the relative volume amount of Si – much stronger than the mere dependence of the conductance from porosity found by Deprez et al.,^43^ who measured the conductance of metals with different porosities and analyzed the data with regard to percolation theory. This indicates that Co incorporation might have a significantly stronger impact on the Si matrix than a simple occupation of active Si sites and blocking the Li transport. Such a behavior could also imply why the Raman signals of Co–Si mixtures shown in Figure 2a are clearly damped even by comparatively small additions of Co.

Thus, the three open questions from the beginning, the low slope for the capacity change with cobalt addition for silicon contents near 100%, the S-shaped curve, and the residual capacity for higher cobalt concentrations (Figure 1), can be unambiguously described by applying a directed percolation model.

## Summary

A material library of cobalt–silicon materials with a continuous compositional gradient was prepared by magnetron sputtering as a high throughput synthesis method, and extensive Raman and X-ray diffraction analyses employing high throughput techniques were used to obtain information about crystal phase development. Cobalt silicides, CoSi_2_ and CoSi, do form under the chosen experimental conditions (in particular, sputtering without external heating of the substrate), but only in a very small compositional range around ratios of 1:1 and 1:2, respectively, and solely to a small extent.

The Raman signal as a measure of lattice vibrations is significantly and continuously changed by the addition of cobalt atoms to the amorphous silicon matrix, even in small amounts (∼10 at %). Therefore, it is concluded that silicon and cobalt atoms are not independently located in addition to each other but set up a kind of “lattice” bond by chemical interaction.

More than 150 samples of cobalt–silicon mixtures in the compositional range between 100 at % silicon and 40 at % silicon and 60 at % cobalt were analyzed with regard to their electrochemical capacity for lithium ions in battery cell applications. The reduction in capacity with increasing cobalt content is attributed to a blocking of the pathways of lithium ions toward unreacted silicon, which is described qualitatively within a percolation model, and an amorphous Co–Si matrix with a smaller capacity for lithium ion storage. Thus, cobalt silicides that emerge and again vanish with composition in very confined regions of the phase diagram composition are not regarded as the origin of the observed monotonous capacity reduction with increasing cobalt content in the electrodes, while the chosen percolation model unambiguously explains all qualitative details of the observed capacity development.
